# Urethane anesthesia depresses activities of thalamocortical neurons and alters its response to nociception in terms of dual firing modes

**DOI:** 10.3389/fnbeh.2013.00141

**Published:** 2013-10-10

**Authors:** Yeowool Huh, Jeiwon Cho

**Affiliations:** ^1^Center for Neural Science, Korea Institute of Science and TechnologySeoul, South Korea; ^2^Neuroscience, University of Science and TechnologyDaejeon, South Korea

**Keywords:** urethane, thalamus, nociception, anesthesia, bursting activity, tonic activity, single unit recording

## Abstract

Anesthetics are often used to characterize the activity of single neurons *in vivo* for their advantages such as reduction of noise level and convenience in noxious stimulations. Urethane has been a widely used anesthetic in thalamic studies under the assumption that sensory signals are still relayed to the thalamus under urethane anesthesia and that thalamic response would therefore reflect the response of the awake state. We tested this assumption by comparing thalamic activity in terms of tonic and burst firing modes during “the awake state” or under “urethane anesthesia” using the extracellular single unit recording technique. We first tested how thalamic relay neurons respond to the introduction of urethane, and then tested how urethane influences thalamic discharges under formalin-induced nociception. Urethane significantly depressed overall firing rates of thalamic relay neurons, which was sustained despite the delayed increase of burst activity over a 4 h recording period. Thalamic response to nociception under anesthesia was also similar overall except for the slight and transient increase of burst activity. Overall, results demonstrated that urethane suppresses the activity of thalamic relay neurons and that, despite the slight fluctuation of burst firing, formalin-induced nociception cannot significantly change the firing pattern of thalamic relay neurons that was caused by urethane.

## Introduction

The sensory thalamus is known to relay sensory information from the spinal cord to the cortex (Jones, 1985). Thus, it has been extensively studied for its sensory gating mechanism, including sleep and pain transmission (Livingstone and Hubel, 1981; Steriade et al., 1993; Kim et al., 2003; Huang et al., 2006; Huh et al., 2012). Early studies suggested the relay function of the thalamus by demonstrating the increased burst activity and relative reduction of tonic discharges of thalamo-cortical (TC) neurons during sleep (Livingstone and Hubel, 1981; Steriade et al., 1993). Accordingly, burst firing has been implicated in blocking the transmission of sensory information while tonic firing has been implicated in relaying sensory information to the sensory cortex (McCormick and Feeser, 1990). In particular, the dual firing capability of a TC neuron has been implicated as a fundamental mechanism of sensory relay function of the thalamus and intrinsic properties of a TC neuron for dual firing mode has been demonstrated by slice electrophysiological studies (Jahnsen and Llinas, 1984; Destexhe et al., 1996, 1998). Furthermore, a recent study demonstrated the interactive relationship between tonic and burst firings of TC neurons in response to nociception using behaving mice, which showed directly proportional changes of tonic firing and inversely proportional changes of burst firing during the awake state (Huh et al., 2012).

Due to the technical nature of behavioral single unit recordings and the assumption that sensory information still reaches the thalamus even under anesthesia (Maggi and Meli, 1986), the firing pattern of thalamic neurons in response to a specific sensory event has been often characterized under anesthesia. Using anesthetized animals would be beneficial in characterizing the specific pattern of neuronal discharge in relation to a certain sensory event because it would be efficient to establish the specific correlation between thalamic responses and a specific sensory event without the influence of other motor and/or sensory events that occur during the awake state. The TC circuit is composed of mostly reciprocal projections between the sensory TC nucleus, reticular thalamic (RT) nucleus and sensory cortex (Jones, 1985). Since the GABAergic neuronal connections from the RT nucleus to the TC nucleus were demonstrated to influence TC neuronal bursting activity in behaving mice (Halassa et al., 2011), the interactions between the RT and TC nuclei could be abnormally potentiated by GABA potentiating-anesthetics, which in turn might alter or distort the patterns of neuronal discharges in response to sensory events. While some studies suggested urethane to be safe for usage in *in vivo* studies to describe the firing pattern in relation to a specific sensory stimulus (Maggi and Meli, 1986), other studies have provided evidences that urethane could also exert significant effects on various neurotransmitter systems including the GABAergic system (Hara and Harris, 2002) and alter striatal discharge patterns in response to sensory stimulations compared to the response in awake animals (West, 1998).

However, there has been no study demonstrating the effect of urethane on thalamic neuronal discharges that can be compared to the firing pattern during the awake state in terms of dual firing modes. The present study investigated the direct effect of urethane anesthesia on neuronal burst and tonic firings in the ventrobasal complex, composed of the ventro-posterior medial (VPM) and ventro-posterior lateral (VPL) nuclei, which is the somatosensory relay thalamus in rodents. Furthermore, thalamic responses to formalin-induced nociception, suggested to be an appropriate clinical pain model (Dubuisson and Dennis, 1977), under urethane anesthesia were also demonstrated and compared to the previous study from this laboratory to investigate the influence of urethane anesthesia on nociceptive signaling in the TC neurons (Huh et al., 2012). The formalin pain model triggers the behaviorally characteristic 1^*st*^ and 2^*nd*^ phase nociceptive responses, respectively corresponding to the acute stimulation of nociceptors and the development of inflammation (Le Bars et al., 2011).

## Materials and methods

### Ethics statement

Procedures for animal use were approved by the Institutional Animal Care and Use Committee of Korea Institute of Science and Technology (protocol number: AP-2011L7006). All surgery was performed under zoletil anesthesia and experiments were carefully conducted to minimize the suffering of mice.

### Animals

First generation hybrids of C57BL/6J × 129/SvJae male mice (8–12 week, body weight 25–30 g) were used in the experiment. Mice had free access to food and water and maintained under a 12 h light/dark cycle (lights on at 8 AM).

### Surgery

Mice were anesthetized with zoletil (30 mg/kg) and fixed on a stereotaxic instrument (David Kopf Instruments, USA) for electrode implantation surgery. Supplementary doses, one-third of the first injection, were given to maintain sufficient level of anesthesia. A small hole in the skull was drilled above the target region to implant a microdrive (Neuralynx, USA) with four tetrodes (nichrome polyamide-insulated microwires, Kanthal precision technology, Sweden; tip gold plated to 400–500 kΩ at 1 kHz) into the sensory thalamus (VPM and VPL; AP: −1.58, ML: −1.8, DV: −3.25). The microdrive was secured onto the skull with stainless steel screws and dental cement. After the implantation surgery, mice were allowed to recover for a week. During the recovery period, mice were handled and habituated to the experimental setting for approximately 20 min every day to minimize stress.

### Extracellular single unit recording

The recording room was equipped with dim lighting and a white noise generator set at the maximum 85 dB. Each mouse was allowed to habituate to the experimental setting for approximately 30 min. Neuronal signals were obtained with the Cheetah Acquisition System (Neuralynx, USA). Signals were filtered, amplified and sampled at 30,303 Hz. For the experiment that tested the effect of urethane on thalamic discharge patterns, the awake state was measured for 10 min and then neuronal activity was measured for four more hrs immediately after intraperitoneal (IP) injection of 1.5 g/kg urethane (awake 10 min + urethane 4 h). For the experiment that tested how nociceptive stimuli would affect thalamic neuronal activity under anesthesia, neuronal discharges were obtained for 10 min during awake state and then recorded for 20 min after urethane (1.5 g/kg) was injected. After the 20 min recording following urethane injection, 10 μl of 5% formalin (1:20 dilution of 37% formalin solution in double de-ionized water) was injected into the contralateral paw of the electrode implantation site and neuronal activity was continuously recorded for an additional hrs (awake 10 min + urethane 20 min + formalin injection under urethane 60 min). No saline injection control was done because previous studies showed that saline injection does not induce any long-term behavioral expression of nociception and does not have a prolonged effect as formalin injections do (Huang et al., 2006; Huh and Cho, 2013). Mice were unrestrained and freely moving during all awake recording sessions. For anesthesia, mice were briefly restrained for IP injection. Mice became fully anesthetized quickly following the urethane injection (within 1 min) when checked by absence of flexion reflex to pinching of the hind paw with forceps. Once anesthetized, mice were laid on a bed of paper towels to prevent body temperature loss. Body temperature was found to be constant for 4 h while animals were under anesthesia (data not shown). For all experiments, only a single injection of urethane was given to each mouse. A single injection was sufficient to keep mice anesthetized for the whole recording period and mice did not show any flexion reflex when checked at the end of the recording period. Neuronal activities were recorded from the right hemisphere while formalin was introduced to the left paw for all experiments. No reflexive behavior was present in response to formalin injection under urethane anesthesia.

### Data analysis

Spike data collected with the Cheetah Acquisition System (Neuralynx, USA) was sorted into single units using the SpikeSort3D program (Neuralynx). Isolated signals were confirmed to originate from a single unit with inter-spike-interval histograms and cross-correlation. Only the well-isolated signals confirmed to be in the ventrobasal complex were used for data analysis. All the signals obtained from the ventrobasal complex were assumed to be from the projection neurons because it has been suggested that no interneurons are present in the thalamic nuclei except in the lateral geniculate nucleus in rodents (Arcelli et al., 1997). Spike trains of individual single neurons were analyzed by firing rate of overall, tonic, and burst in 5 min segments. Tonic and burst spikes were separated by distinguishing burst spikes with the following criteria: spikes consisting of at least two spikes occurring within ≤ 4 ms with ≥ 100 ms proceeding silence (Lu et al., 1992). Non-burst spikes were considered to be tonic spikes. Along with the firing rate analysis, bursting properties—changes of burst spike proportion over time, interval between bursts, length of burst, interval between burst spikes (IntraBI) and the number of burst spikes composing a burst—were also analyzed over time in 5 min segments. Details on how the burst property analysis was performed are delineated in Figure 2A. To compare the change of firing rate relative to the awake or urethane anesthetized state of individual cells after formalin injection, data were normalized by the following method: (firing rate after formalin injection—awake or urethane baseline firing rate) / (firing rate after formalin injection + awake or urethane baseline firing rate). This normalization method gives an accurate representation of the neural changes induced by formalin relative to the each individual cell baselines, but the magnitude of neuronal changes is not reflected by this method. The awake baseline is the average of two 5 min segments of the spontaneous neuronal activity during the awake state while the urethane baseline is the average of two 5 min segments recorded 10–20 min after urethane injection. This normalization method also enabled us to compare current results with those of awake results that were reported previously (Huh et al., 2012) in terms of thalamic responses in response to the formalin injection.

### Statistical analysis

Repeated measures ANOVA was used to test for changes in firing rate and bursting properties over time after urethane in the “urethane only” experiment and after formalin injection in the formalin injection under urethane anesthesia experiment. Student’s *t*-test was used to test for changes induced by urethane injection relative to the awake baseline in the “urethane only” experiment. Changes induced by formalin under urethane anesthesia were tested by using the student’s *t*-test between the urethane baseline (10–20 min after urethane injection) and after formalin injection. A *p*-value of 0.05 was used to determine significance.

### Histology

After completion of the entire experiment, locations of the recording sites were verified post mortem. Mice were overdosed with 2% avertin. Once mice were fully anesthetized, electrolytic lesions were made by passing 5–30 μA current for 10 sec through the recording electrode. Afterwards, mice were transcardially perfused with saline and then with 10% formalin diluted in saline. Brains were extracted and further fixed in 10% formalin solution for a day and stored in 20% sucrose solution for a week before sectioning. Coronal sections of 50 μm were cut through the thalamic formation using the microtome cryostat (Microm, Germany). The sections were stained with Cresyl Violet (Sigma, USA) and examined under a light microscope to determine the recording sites.

## Results

### Urethane suppresses neuronal discharges in the sensory thalamus

**Figure 1:**
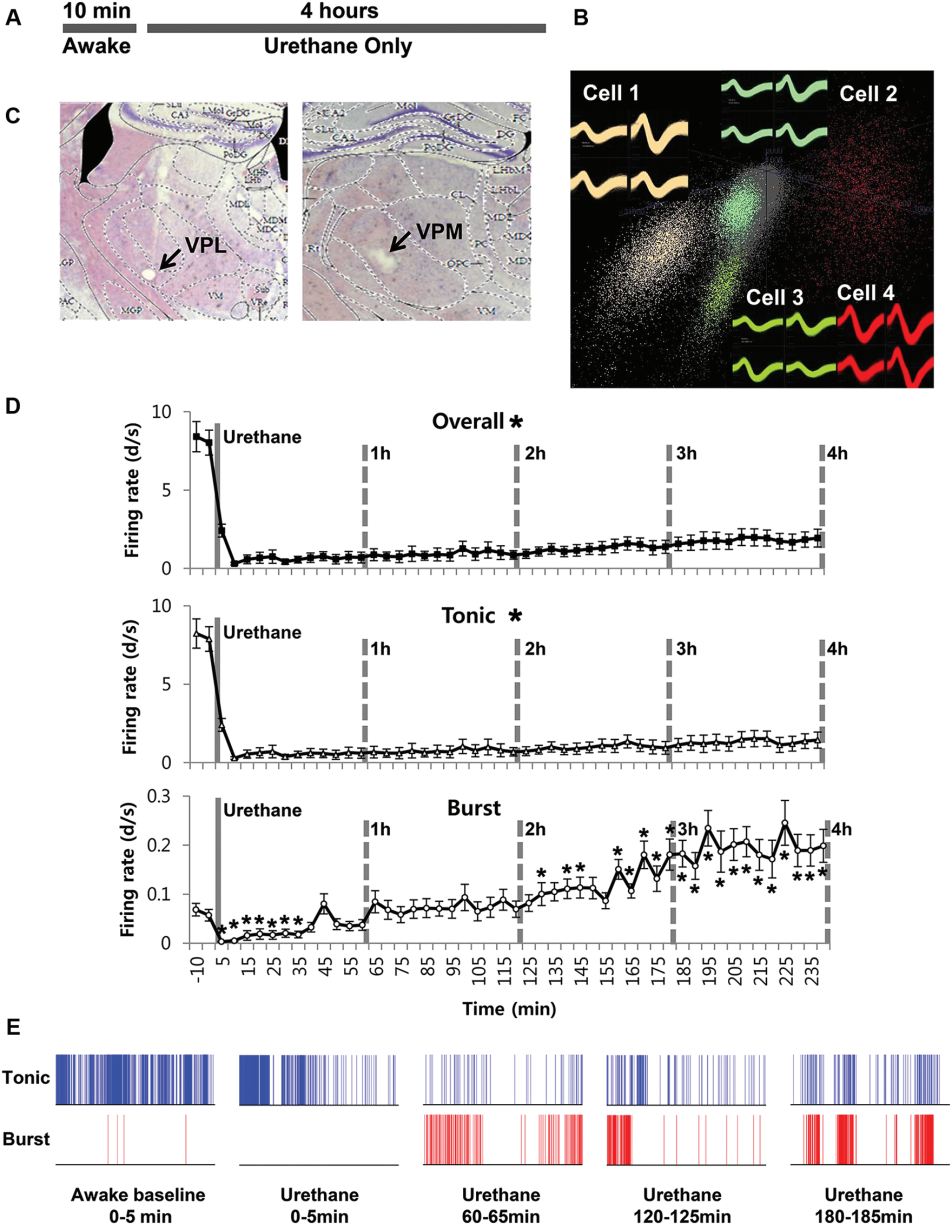
**Thalamic neuronal activity changes by urethane anesthesia**. **(A)** Timeline of the experimental procedure. **(B)** Spike sorting sample of multi-spike data from one tetrode into four single units using the SpikeSort3D program, which allows spike sorting based on the differences in waveform shape captured by the four individual electrodes composing a tetrode. **(C)** Histological sample of the recording locations. **(D)** Firing rate changes for overall, tonic and burst firing rate of thalamic neurons during awake and under urethane anesthesia analyzed in 5 min segments. Repeated measures ANOVA was used for statistical analysis over time. *N* = 32 neurons, 7 mice. All data points are mean ± SEM. Solid vertical gray-line indicates the point of urethane injection while the dotted vertical gray-line was placed to facilitate the visualization of firing pattern change over time after urethane anesthesia. Student’s *t*-test was used to compare before and after urethane. * Indicates significant difference at *p* < 0.05. When all points were significantly different from the awake baseline, * was placed next to the title. There was significant change over time for overall, tonic, and burst firing after urethane injection when repeated measures ANOVA analysis was used. **(E)** Spike train sample of tonic and burst firing from one neuron, in 5 min segments as indicated.

In order to investigate the effect of urethane alone on TC neurons in the ventrobasal complex, individual neuronal discharges were observed for 4 h following urethane injection (1.5 g/kg). All the neurons analyzed in this study were TC projection neurons, since interneurons are absent in the thalamic nuclei of rodent except in the lateral geniculate nucleus (Arcelli et al., 1997). The experimental procedure is delineated in Figure 1A. Thirty-two single neurons from 7 mice, which were isolated by spike sorting methods (Figure 1B, see Section Materials and Methods for details), were analyzed after histological verification (Figure 1C).

Urethane-induced changes were abrupt, profound and long-term, continuously sustained during the entire recording period of 4 h. Only urethane was injected for the “urethane only” experiment. The average overall firing rate of TC neurons before urethane injection was 8.2 spike discharges per second (d/s). However, the average firing rate was dramatically and significantly reduced to 0.3 d/s within 10 min after urethane injection, slowly increased to 1.94 d/s during the 4 h after the injection, but never recovered to the baseline firing rate of the awake state (Table 1).

**Table 1:** **Thalamic neuronal activity before and after urethane anesthesia (“urethane only”)**.

	**Baseline (Awake)**		**Urethane 1h**		**Urethane 2h**		**Urethane 3h**		**Urethane 4h**	
	**FR (d/s)**	**Ratio (%)**	**FR (d/s)**	**Ratio (%)**	**FR (d/s)**	**Ratio (%)**	**FR (d/s)**	**Ratio (%)**	**FR (d/s)**	**Ratio (%)**
Overall	8.21 ± 0.19		0.77 ± 0.15		0.93 ± 0.05		1.27 ± 0.06		1.94 ± 0.04	
Tonic	8.06 ± 0.17	98.1	0.70 ± 0.16	90.3	0.75 ± 0.04	80.4	0.98 ± 0.05	77.2	1.44 ± 0.04	73.0
Burst Spike	0.15 ± 0.02	1.9	0.06 ± 0.01	9.7	0.18 ± 0.01	19.5	0.29 ± 0.24	22.8	0.20 ± 0.01	27.0

Baseline is the spontaneous neural activity of the awake state before urethane injection. Neuronal activity after urethane injection was analyzed in 1 hrs intervals. *N* = 32 neurons, 7 mice. All values are mean ± SEM. FR: firing rate, d/s: discharges/sec, ratio: percentage of respective firing modes from the total number of spikes. No formalin was injected at any point.

To see specific effects of urethane on burst and tonic firings, burst and tonic firings were separated from the spike train of individual neurons (spikes consisting of at least two spikes occurring within ≤ 4 ms with ≥ 100 ms preceding silence were considered to be burst spikes while non-burst spikes were considered to be tonic; Lu et al., 1992). First, the effect of urethane injection on tonic firing was similar to that for the overall firing rate. Thus, urethane injection immediately dropped the average tonic firing rate to 0.3 d/s within 10 min from the baseline firing rate of approximately 8 d/s, then gradually but continuously recovered to 0.7 d/s during the 1^*st*^ h, 0.75 d/s during the 2^*nd*^ h, 0.98 d/s during the 3^*rd*^ h and 1.44 d/s during the 4^*th*^ h after the urethane injection (Table 1). Despite the continuous augmentation over time, tonic firing rate never significantly recovered to the baseline during the 4 h of the recording period (Figure 1D, Tonic).

The temporal changes of burst firing induced by urethane were somewhat different. For example, urethane rapidly depressed the burst spike firing rate down to 0.01 d/s from the baseline of 0.15 d/s within 10 min after the injection, but then the average rate was increased to 0.06 d/s during the 1^*st*^ h, 0.18 during the 2^*nd*^ h, 0.29 during the 3^*rd*^ h and 0.20 during the 4^*th*^ h of recording (Table 1). This indicates that bursting activity was eventually potentiated by urethane injection by the end of the 2^*nd*^ h and continued to escalate during the rest of recording period (Figure 1D, Burst). However, the proportion of tonic spikes was always greater than that of burst spikes at all conditions. An example of tonic and burst firing changes in a neuron before and after urethane anesthesia are depicted in Figure 1E.

### Effect of Urethane on Other Bursting Properties

**Figure 2:**
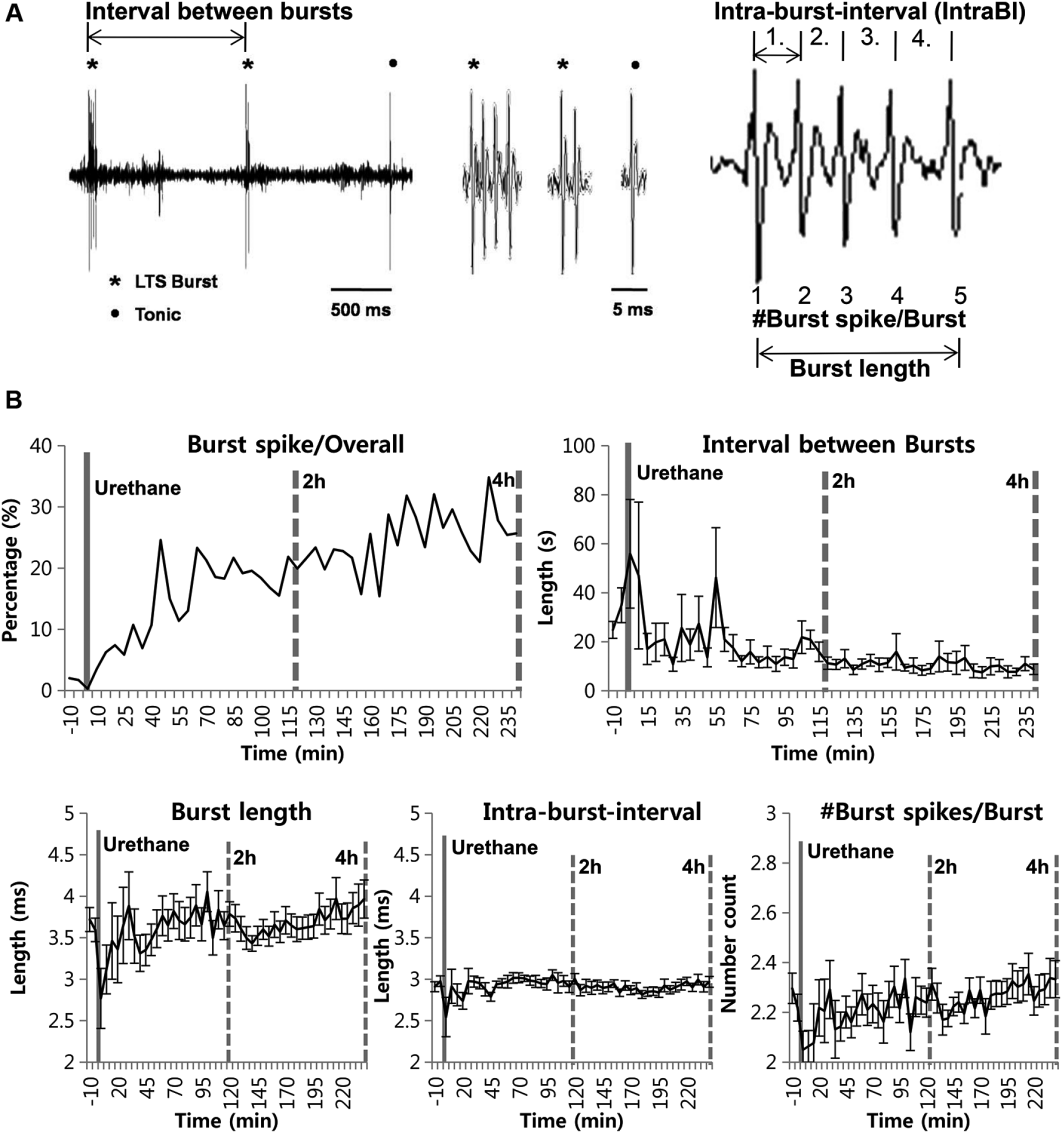
**Urethane anesthesia and thalamic bursting properties**. **(A)** Figurative illustration showing how bursting property analysis was performed. **(B)** Thalamic neuronal burst firing property changes by urethane anesthesia. *N* = 32 neurons, 7 mice. All data points are mean ± SEM, except for the proportion of burst spikes analysis which was calculated based on mean value. Solid vertical gray-line indicates the point of urethane injection while the dotted vertical gray-line was placed to facilitate the visualization of firing pattern change over time after urethane anesthesia. Repeated measures ANOVA was used to test significance over time after urethane injection.

We also investigated whether urethane injection induces any changes in burst firing properties (Figure 2A). Urethane injection changed the proportion of burst spikes, the spikes that constitute bursts, over time. For example, a baseline of 1.88% significantly increased to 9.65% during the 1^*st*^ h after the formalin injection, 19.54%, 22.79% and 26.97% hourly during the 2^*nd*^, 3^*rd*^ and 4^*th*^ h, respectively. However, these increased ratios are mainly due to the greater suppression of tonic firing despite slight increase of burst firing. Accordingly, corresponding to the increased bursting frequency, the mean inter-burst interval gradually decreased over time (Figure 2B).

However, other parameters for bursting properties, such as the burst length, the IntraBI and the number of burst spikes composing a burst did not show any significant changes over time despite some fluctuations (Figure 2B). These results show that urethane gradually, but significantly increased the occurrence of burst activities without influencing several properties of a burst composition.

### Thalamic responses to the formalin-induced nociception under urethane anesthesia

**Figure 3:**
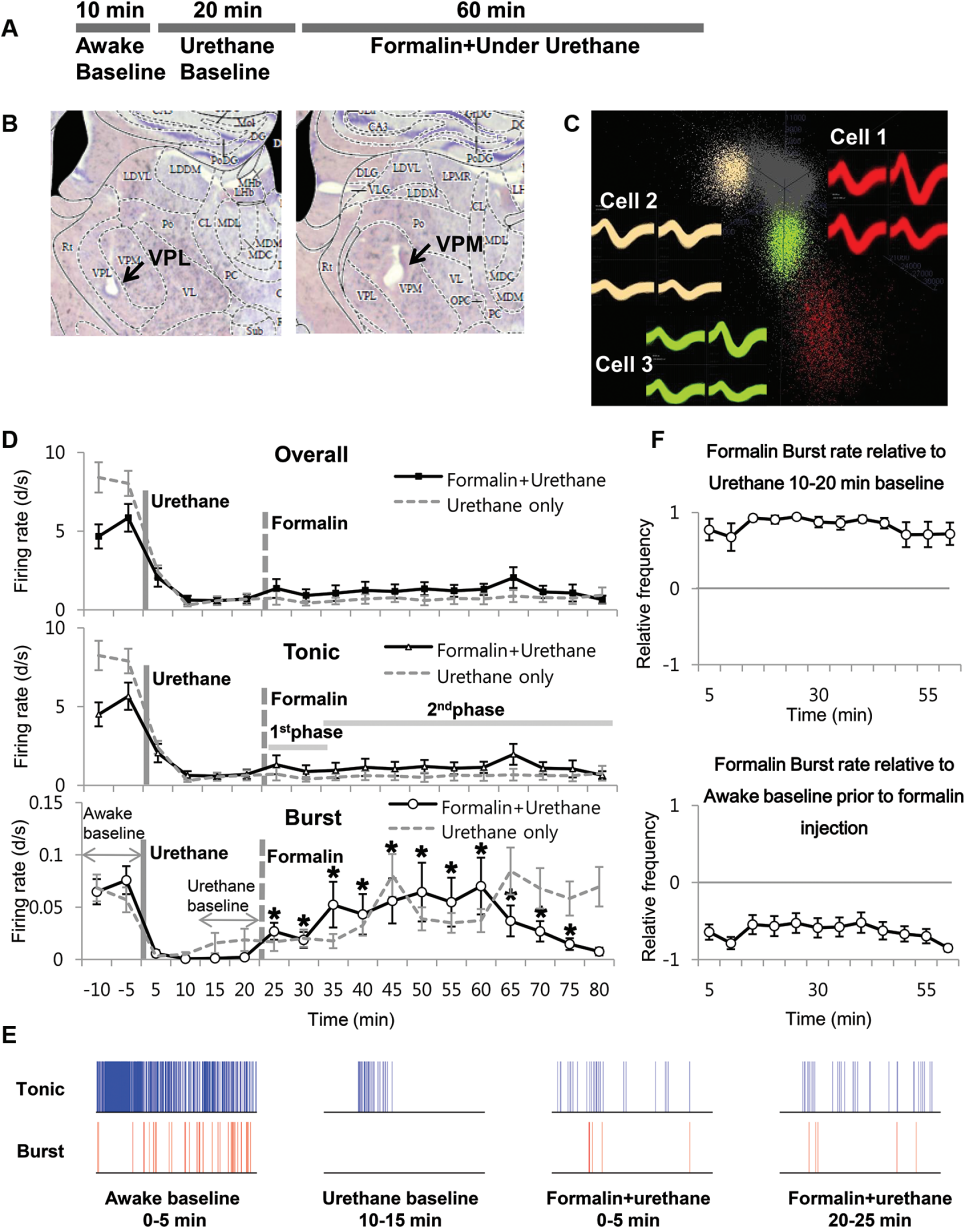
**Formalin-induced activity changes of thalamic neurons under urethane anesthesia**. **(A)** Timeline for experimental procedures. **(B)** Histological sample of the recording locations. **(C)** Spike sorting sample of multi-spike data from one tetrode into three single units with the SpikeSort3D program. **(D)** Firing rate changes for overall, tonic and burst firing rate of thalamic neurons during the awake state, under urethane anesthesia and after formalin injection, juxtaposed with the respective firing rates of “urethane only” experiment from Figure 1 for comparison. Solid vertical gray-line indicates the point of urethane injection while the dotted vertical gray-line indicates the point of formalin injection. The horizontal gray lines marked as 1^*st*^ phase and 2^*nd*^ phase are based on the results of a typical behavioral response of the formalin test, which has the 1^*st*^ and 2^*nd*^ phase nociceptive responses separated by the interphase with low pain response. The horizontal gray arrows indicate the intervals used for normalization in panel E. **(E)** Spike train sample of tonic and burst firing from one neuron, representing the awake, after urethane injection, and 1^*st*^ and 2^*nd*^ phase of after formalin injection under urethane anesthesia in 5 min segments. **(F)** Normalized value of burst firing rate changes due to formalin injection. Awake state or urethane-injected state prior to formalin injection was used as baseline for normalization to emphasize the relative firing rate changes. For urethane baseline, the value for 10 min before formalin injection was used. See Section Materials and Methods for details. (D and E) *N* = 29 neurons, 6 mice. Student’s *t*-test was used to compare each data points with the urethane baseline. *Indicates significant difference at *p* < 0.05.

Next, we investigated the thalamic responses to the formalin-induced nociception under the influence of the urethane anesthesia to see how TC neurons under urethane respond to the nociceptive sensory stimulation as outlined (Figure 3A). A total of 29 neurons, isolated by spike sorting method, from another set of mice (*n* = 6) were included in the analysis after histological verifications (Figure 3B). Formalin was injected intradermally 20 min after the urethane (1.5 g/kg) injection, and thalamic response was recorded for an additional hour; these parameters were chosen specifically so that the results could be compared with the results of a previous study (Huh et al., 2012). As shown in the urethane-alone experiment, average overall firing rate of TC neurons rapidly decreased by urethane injection from 5.25 d/s to 1.14 d/s during the 10–20 min segment after urethane injection and remained at approximately 1 d/s throughout the recording session even after formalin was injected, indicating that overall firing under the urethane was not influenced by subsequent formalin-induced nociception (Table 2). Likewise both tonic and burst firings rates were also dramatically reduced after the urethane injection. For example, both average tonic and burst firings dropped from 5.08 d/s to 0.64 d/s and 0.017 d/s to 0.001 d/s, 87% and 94% reduction, respectively, during the 10 min after urethane injection (Table 2).

**Table 2:** **Activity of thalamic neurons during the awake state, under urethane anesthesia, and after formalin injection**.

	**Baseline (Awake)**		**Baseline (Urethane) 10-20 min**		**Formalin 1^*st*^ phase (0-10 min)**		**Formalin 2^*nd*^ phase (10-60 min)**	
	**FR(d/s)**	**Ratio(%)**	**FR(d/s)**	**Ratio(%)**	**FR(d/s)**	**Ratio(%)**	**FR(d/s)**	**Ratio(%)**
Overall	5.25 ± 0.80		0.64 ± 0.24		1.14 ± 0.57		1.22 ± 0.14	
Tonic	5.08 ± 0.80	96.65	0.64 ± 0.24	100.00	1.09 ± 0.57	95.73	1.12 ± 0.14	91.99
Burst Spike	0.17 ± 0.03	3.35	0.00 ± 0.00	0.0	0.05 ± 0.52	4.27	0.10 ± 0.01	8.01

Awake baseline is the spontaneous neural activity of the awake state. Urethane baseline is the spontaneous neural activity under urethane anesthesia. Neural response after formalin injection is divided into the 1^*st*^ and 2^*nd*^ phases based on the typical behavioral response of the formalin test which has a quiescent interval between the two peaks of the behavioral pain responses. *N* = 29 neurons, 6 mice. All values are mean ± SEM. FR: firing rate, ratio: percentage of respective firing modes from the total number of spikes.

Interestingly, formalin injection did not induce any changes in the average tonic firing pattern under urethane anesthesia (Figure 3D Tonic), possibly suggesting that thalamic neurons were not relaying any nociceptive information. The average tonic firing rate remained at approximately 1 d/s, which was not significantly different from that of the urethane baseline. The average burst firing, on the other hand, drastically increased from 0.002 d/s to 0.023 d/s within 5 min after formalin injection and remained significantly elevated above the baseline burst firing level of urethane (Figure 3D Burst). Formalin injection, under urethane anesthesia, caused immediate and rapid increase of burst firing, which was more potentiated and sustained between the 10–15 min and 35–40 min segments after formalin injection (respectively corresponding to 30–35 min and 55–60 min segments after urethane injection). The burst firing rate tended to decrease after 45 min following formalin injection, but it was still significantly above the urethane baseline level. Representative sample of tonic and burst firing changes from one neuron for awake, after urethane injection, and formalin injection under urethane anesthesia are depicted in Figure 3E.

Nonetheless, formalin-induced increase of burst firing was still significantly below the awake burst firing level when normalized (Figure 3F). In this normalization method, a value below zero indicates the reduction relative to the baseline. Taken together, the results show that thalamic responses in terms of tonic and burst firings to formalin under urethane anesthesia is clearly different from the responses during the awake state shown in a previous study that had been performed in the same experimental condition (Huh et al., 2012).

### Thalamic bursting property changes induced by formalin under urethane anesthesia

**Figure 4:**
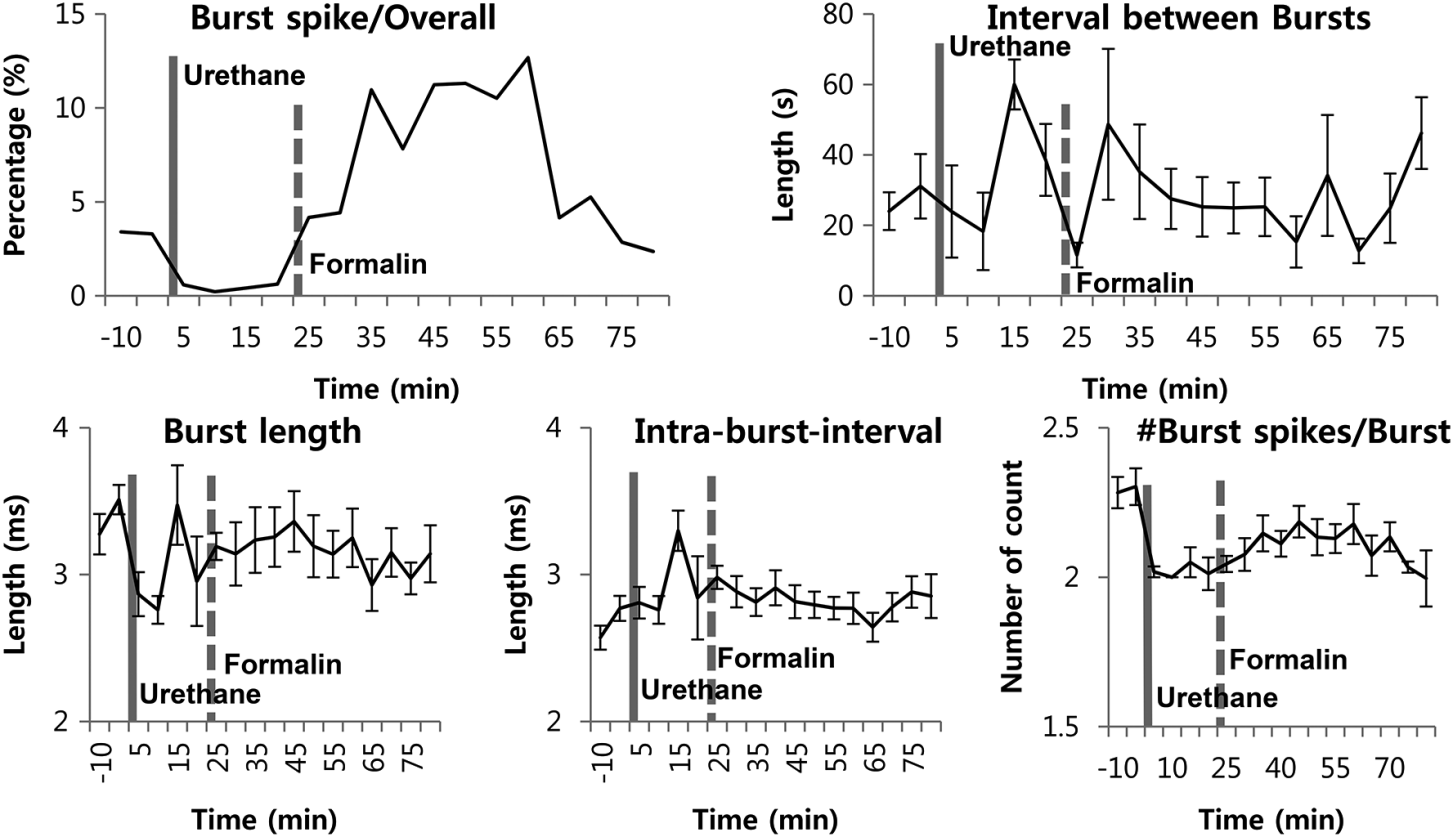
**Thalamic burst properties during the awake state, under urethane anesthesia and after formalin injection under urethane anesthesia**. *N* = 29 neurons, 6 mice. Solid vertical gray-line indicates the point of urethane injection while the dotted vertical gray-line indicates the point of formalin injection. Repeated measures ANOVA was used to test significance over time after formalin injection.

A previous study from our lab has shown that not only the burst firing pattern, but also other bursting properties changed accordingly to the phasic changes of the formalin-induced behavioral responses in the awake state (Huh et al., 2012). Based on this observation, we investigated whether formalin would also induce such bursting property changes under urethane anesthesia. Although the proportion of burst spikes rose and fell in accordance with the increased bursting occurrence induced by formalin, other properties, such as burst spike number per burst, burst length, and IntraBI remained consistent aside from few insignificant initial fluctuations (Figure 4).

Overall, the results suggest that, although transient increase in TC neuronal burst firing can be induced under urethane anesthesia, urethane disrupts dynamic responses of thalamic burst discharges to nociception, as no changes in burst firing properties were induced by formalin injection, unlike in the awake state.

## Discussion

Our results demonstrate the influences of urethane on TC tonic and burst firings of behaving mice and further characterize the thalamic responses to the formalin injection under urethane anesthesia. The results may provide insights to the thalamic mechanism of long-lasting anesthetic effect of urethane. First, the urethane-alone experiment showed that urethane injection completely suppressed tonic firing while bursting was initially depressed and gradually potentiated above the baseline of the awake state. That is, one way that urethane may exert its anesthetic action is by suppressing tonic firing of TC neurons that is known to faithfully carry sensory information to the cortex while potentiating burst firing that is believed to block the sensory information (Kim et al., 2003; Huh et al., 2012). A previous study demonstrated the loyal reflection of tonic firing in correspondence to the behavioral nociceptive responses (1^*st*^ phase 8.60 ± 0.57 d/s, 2^*nd*^ phase 6.29 ± 0.23 d/s) and the opposite phenomenon in burst firing (1^*st*^ phase 0.12 ± 0.02 d/s, 2^*nd*^ phase 0.44 ± 0.03 d/s) of TC neurons with significant changes in burst properties during different behavioral nociceptive phases (Huh et al., 2012). Interestingly, the fact that no significant changes in bursting properties were observed by formalin-induced nociception under urethane anesthesia, aside from the increase in burst firing frequency, may indicate that cortical feedback may be necessary to modulate burst firing properties. That is, suppressed tonic activity and gradually potentiating burst activity may have caused a complete blockade of sensory transmission to the cortex and, consequently, TC neurons could not receive feedback to modulate burst property changes in the anesthetized state. In particular, these changes in thalamic discharges appear to be greatly modulated by GABAergic inhibition induced by urethane over time (Hara and Harris, 2002) because TC neurons receive strong GABAergic projections from the RT. Taken together, these results suggest that urethane maintains the anesthetic state by decreasing tonic activity and gradually potentiating burst activity in the sensory relay thalamus.

Furthermore, the introduction of formalin-induced inflammation to urethane-anesthetized mice has interesting implications. Formalin test is a type of tonic pain model, suggested to be a relevant clinical pain model (Dubuisson and Dennis, 1977), and the current results will provide insights to the possible influence of urethane anesthesia in investigating other tonic pain models. First, tonic firing that was being already suppressed by urethane did not show any response, indicating a behaviorally anesthetized state with complete loss of sensation. However, there was an immediate increase in burst firing in response to formalin injection under anesthesia within a couple of minutes, indicating that the generation of burst firing was somehow triggered by nociceptive sensory activation but not by the abrupt increase of tonic firing as shown in during the awake state (Huh et al., 2012). This is particularly intriguing because burst generation is assumed to be triggered by the relay of sensory signals to the cortex or the RT by the post-synaptic actions of tonic firing of TC neurons, and further suggests the presence of other pathways to the RT or cortex to begin the generation of TC bursting. Another possibility is that TC bursting under urethane acts as a detector of the incoming nociceptive inputs as reported in the visual system and the sensory system (Fanselow et al., 2001; Sherman, 2001), suggesting a completely different role of TC bursting during anesthetized state from during the awake state. Yet another possibility is that the increase in burst firing could be the effect of urethane anesthesia, since urethane injection caused burst firing to constantly increase over time. It is worth noting that formalin injection under urethane quickly potentiated burst activity, which was supposed to gradually increase over time in the “urethane only,” and that there was no interactive relationship between tonic and burst firings under urethane. Most importantly, our results show that firing patterns in response to formalin injection under urethane anesthesia are completely different from those of the awake state (Huh et al., 2012). For example, unlike the results of the previous study (Huh et al., 2012), tonic firing in response to formalin injection under urethane did not show any temporal modulations corresponding to the behavioral phase of the formalin task during the awake state. On the contrary, burst firing showed clear and immediate response to formalin injection for the entire recording session, again suggesting that nociceptive signals still reach the thalamus even under the influence of urethane. Furthermore, burst properties, including IntraBI and others, were not influenced, probably indicating that burst response to formalin under urethane contributes to the assured blockade of remaining sensory transmission to the sensory cortex. It is unclear at the moment why potentiated burst activity is required in addition to the severely suppressed tonic activity to block nociceptive signals induced by formalin. However, since thalamic responses under anesthesia and during the awake state are different, investigating behavioral correlations with thalamic responses is probably unsuitable under urethane anesthesia.

In summary, urethane significantly depresses both tonic and burst firing activities of thalamic neurons, and the response of thalamic neurons to formalin-induced nociception under urethane is different from that of the awake state.

## Author contributions

Both authors had full access to all data and take responsibility of the presented data. Study design, data analysis, and drafting of the manuscript were done by both authors. All data were acquired by Yeowool Huh and the study was supervised by Jeiwon Cho.

## Conflict of interest statement

The authors declare that the research was conducted in the absence of any commercial or financial relationships that could be construed as a potential conflict of interest.
